# Role of Sleep Restriction in Daily Rhythms of Expression of Hypothalamic Core Clock Genes in Mice

**DOI:** 10.3390/cimb44020042

**Published:** 2022-01-25

**Authors:** Weitian Li, Zixu Wang, Jing Cao, Yulan Dong, Yaoxing Chen

**Affiliations:** Neurobiology Laboratory, College of Veterinary Medicine, China Agricultural University, Beijing 100193, China; l752390753@163.com (W.L.); zxwang2007@163.com (Z.W.); caojing315@126.com (J.C.); ylbcdong@cau.edu.cn (Y.D.)

**Keywords:** circadian rhythm, clock gene, sleep restriction, hypothalamic

## Abstract

Lack of sleep time is a menace to modern people, and it leads to chronic diseases and mental illnesses. Circadian processes control sleep, but little is known about how sleep affects the circadian system. Therefore, we performed a 28-day sleep restriction (SR) treatment in mice. Sleep restriction disrupted the clock genes’ circadian rhythm. The circadian rhythms of the *Cry1* and *Per1/2/3* genes disappeared. The acrophase of the clock genes (*Bmal1, Clock, Rev-erbα*, and *Rorβ*) that still had a circadian rhythm was advanced, while the acrophase of negative clock gene *Cry2* was delayed. Clock genes’ upstream signals ERK and EIFs also had circadian rhythm disorders. Accompanied by changes in the central oscillator, the plasma output signal (melatonin, corticosterone, IL-6, and TNF-α) had an advanced acrophase. While the melatonin mesor was decreased, the corticosterone, IL-6, and TNF-α mesor was increased. Our results indicated that chronic sleep loss could disrupt the circadian rhythm of the central clock through ERK and EIFs and affect the output signal downstream of the core biological clock.

## 1. Introduction

Most people cannot reach the recommended sleep time [1,2]. Lack of sleep causes extensive harm to human health, including metabolic function, emotional regulation, and adverse effects on cognitive function [3,4,5,6]. Sleep regulation includes sleep pressure (process S) and the circadian rhythm (process C). As waking time increases, sleep pressure increases, leading to sleep. The circadian rhythm controls the transition between sleep and wakefulness [7]. However, it is unclear how increased sleep pressure caused by reduced sleep time affects the circadian rhythm. Research on this issue can help us to understand how a lack of sleep damages the body and to find ways to alleviate and improve it.

The central biological clock of mammals is located in the hypothalamus’s suprachiasmatic nucleus (SCN). The circadian clock system has three main systems. The first is the negative feedback loop composed of aryl hydrocarbon receptor nuclear translocator (BMAL1)/CLOCK and PER/CRY. BMAL1/CLOCK promotes the transcription of *Period* (*Per*)/*Cryptochrome* (*Cry*), and elevated PER/CRY inhibits the transcription of *Bmal1*/*Clock* [8]. At the same time, PER/CRY is not stable and degrades over time. When it degrades, the transcription of *Clock*/*Bmal1* restarts, thus forming a loop [9,10]. The nuclear receptor subfamily 1 (REV-ERBα), which rhythmically inhibits the transcription of *Bmal1* and nuclear factor interleukin-3 regulation (*Nfil3*), is driven by the activation of retinoic acid-related orphan receptors (RORs) in this process [11,12]. Second, BMAL1/CLOCK is also regulated by REV-ERBs and RORs [13]. NFIL3, in turn, inhibits the alkaline leucine zipper rich in proline and acidic amino acids (PAR-bZip) factor D-box binding protein (DBP) to regulate the rhythm of the ROR nuclear receptor [14]. The third transcription loop driven by CLOCK-BMAL1 involves PAR-bZip factor DBP, thyroid stimulating hormone embryonic factor (TEF), and hepatic leukemia factor (HLF) [10]. These protein interactions contain the site D-box, repress NFIL3, and are driven in association with the REV–ERB–ROR cycle [15].

Previous studies showed that a reduction in sleep time affected the expression of clock genes, including a decrease in amplitude and changes in DNA methylation [16,17,18]. At the same time, it caused the malfunction and weakening of the clock protein translation process [19,20]. However, compared to total sleep deprivation with no sleep for a short time, long-term chronic sleep time loss caused by work and illness is more common. The sleep restriction model is more suitable for the actual situation [21]. These studies also rarely describe time changes of the clock gene. Therefore, it is necessary to explore the effect of long-term sleep restriction on the core biological clock rhythm.

Various types of signals guide the circadian rhythm of mammals, including optical and non-optical signals. The extracellular signal-regulated protein kinase (ERK) pathway is involved in various signal-mediated regulations of the biological clock rhythm [22]. The ERK pathway is involved in the phase shift caused by lack of sleep in Syrian hamsters [23]. Phosphorylated ERK can regulate the expression and activity of nuclear factor erythroid 2-related factor 2 (NRF2) and regulate the biological clock through NRF2, integrating cellular redox signals into circadian timing [24,25]. Sleep restriction is a non-optical signal [22,26,27]. At the same time, the ERK pathway leads to the phosphorylation of eukaryotic translation initiation factor 4E (EIF4E) and facilitates light-induced PER protein synthesis [28]. Eukaryotic translation initiation factor 2α (EIF2α), which plays an important role in endoplasmic reticulum stress, regulates the transcription and translation of SCN’s biological clock by receiving optical signals [29]. Exploring changes in the upstream signal of the biological clock in the SR could help us to understand the mechanism of rhythm disturbances caused by long-term lack of sleep.

Mammalian SCN regulates various physiological functions of the body by outputting signals, among which melatonin and corticosterone are crucial. The hypothalamic SCN strictly controls its synthesis. At the same time, melatonin is an essential light-dependent sleep regulator [30]. Through its influence on the hypothalamic–pituitary–adrenal axis, SCN regulates the circadian secretion of corticosterone [31]. Melatonin and corticosterone can change the circadian rhythm of gene expression in peripheral tissue and are essential signals that guide the rhythm of peripheral tissue [32]. Interleukin 6 (IL-6) and TNF-α are closely related to circadian rhythm and sleep. IL-6 is a mediator of sleepiness, and its circadian rhythm pattern reflects the steady-state drive of sleep [33]. TNF-α is a cytokine that induces fatigue, which is elevated in the disease of excessive daytime sleepiness [34]. Therefore, exploring the rhythmic changes of these output signals after sleep restriction is greatly significant for understanding how the output signal of the biological clock is affected by the reduction in sleep time.

In this study, we established a 28-day sleep restriction mouse model. Moreover, we detect the circadian rhythm of clock genes in the hypothalamus and plasma rhythm signal. We describe the time pattern of core clock genes in the hypothalamus of control mice and analyze the effect of reduced sleep time on the expression of these clock molecules and rhythm signals in plasma. Our findings suggest that chronic sleep deprivation impairs the circadian rhythm of the hypothalamus, which in turn leads to disturbances in the rhythm of circadian clock output signals, causing damage to the body.

## 2. Materials and Methods

### 2.1. Experimental Animal Treatments and Ethics Statement

All experiments were conducted following the Guide for the Care and Use of Laboratory Animals published by the Animal Welfare Committee of the Agricultural Research Organization, China Agricultural University (approval no. AW18079102-2). The origin, sex, and adaptation process of animals were the same as those described earlier [35]. Briefly, male ICR mice (8 weeks of age; Vital River Laboratory Animal Technology Co. Ltd., Beijing, China) were prepared for the experiment. After a week of adaptation, mice were divided into the sleep restriction (SR) and control (CON) groups.

The device and method of sleep restriction were the same as we used before [36]. The sleep restriction of the mice was from 12:00 p.m. to 8:00 a.m. the following day, and the sleep deprivation treatment was canceled within 4 h from 8:00 a.m. to 12:00 p.m. The treatment lasted 4 weeks. We set light-on time 7:00 a.m. as ZT0.

### 2.2. Sample

We collected samples from 8:00 a.m. on the 29th day. Mice were euthanized under anesthesia using 10% chloral hydrate every 4 h, and their hypothalamus and blood were harvested. In total, plasma and hypothalamus were collected at 6 time points (8:00, 12:00, 16:00, 20:00, 0:00, 4:00). After collecting the hypothalamus, we froze it in liquid nitrogen and stored it at −80 °C. The blood was anticoagulated with heparin in saline and centrifuged, and the plasma was stored at −80 °C for subsequent hormone measurement.

When collecting materials from the mouse hypothalamus, we first took out the mouse’s whole brain and turned it upside down. At that time, there was a clear frontal optic chiasm and a posterior mamillary body. Between the two was the position of the hypothalamus. After determining the position of the hypothalamus, we removed it along the border to obtain the entire hypothalamus.

### 2.3. Enzyme-Linked Immunosorbent Assay

We tested melatonin, corticosterone, IL-6, and TNF-α in plasma using a previously described method [36]. In short, we used a competitive enzyme-linked immunosorbent assay (ELISA) (Uscn Life Science, Inc., Wuhan, China). The operation was performed following the instructions provided in the kit. Each sample was tested in triplicate.

### 2.4. Real-Time Reverse Transcription Polymerase Chain Reaction (qRT-PCR)

We used a previously described method [37]. In short, we first extracted the mRNA of the mouse hypothalamus and then performed reverse transcription to obtain the cDNA. Then, clock gene primers were designed: *Bmal1*, *Clock*, *Cry1*, *Cry2*, *Per1*, *Per2*, *Per3*, *Rorβ*, *Rev-erbα*, and *Gapdh*. Then, we used these primers to perform a real-time quantitative PCR experiment and used *Gapdh* as an internal reference to express the expression level of each gene. Experiments were repeated in triplicate. PCR primers are listed in Table 1.

### 2.5. Western Blotting

We extracted hypothalamic tissue protein and the Western blot test according to the previously described method [37]. In this part of the experiment, we used the following antibody concentrations: β-actin, 1:8000 (Proteintech, 66009-1-Ig); β-tubulin, 1:5000 (Abmart, M20005); PER2, 1:3000 (Boster, PB0347); ERK, 1:8000 (Sigma, M5670); p-ERK, 1:8000 (Sigma, M8159); NRF2, 1:1000 (Boster, PB9290); p-EIF4E, 1:2000 (Novus Biologicals, NBP266802); p-EIF2α, 1:1000 (Thermo Fisher Scientific, MA5-15133).

### 2.6. Statistical Analysis

We used a previously described method [37]. Briefly, we used the method of cosine fitting to determine whether the data had a 24 h circadian rhythm. First, we performed one-way ANOVA on the data at different time points to determine significant differences between different time points. Then, we added the data into the formula: y = a + b×cos(x×pi/12-c×pi/12). We could calculate the correlation coefficient of determination (R^2^) of the data under this formula and the values of the three parameters of mesor, amplitude, and acrophase. After that, R^2^ was tested using free F and *p*-values. If the *p*-value was less than 0.05, this dataset was considered to have had a circadian rhythm of 24 h.

Differences between groups were statistically analyzed using *t*-tests; *p*-values less than 0.05 were considered indicative of statistical significance. Calculations were performed with Graphpad Prism 8.0.2 (GraphPad Software Inc., San Diego, CA, USA).

## 3. Results

### 3.1. Circadian Rhythm Expression of Mice Hypothalamic Clock Genes in CON Group

We first detected the daily time pattern of nine core clock genes (*Bmal1*, *Clock*, *Cry1*, *Cry2*, *Per1*, *Per2*, *Per3*, *Rorβ*, and *Rev-erbα*) in the hypothalamus of mice in the CON group. All clock genes showed evident diurnal oscillations over time (*Bmal1*: F (_5, 12_) = 3.809, *p* = 0.0268; *Clock*: F (_5, 12_) = 7.899, *p* = 0.0017; *Cry1*: F (_5, 12_) = 9.377, *p* = 0.0008; *Cry2*: F (_5, 12_) = 3.889, *p* = 0.0250; *Per1*: F (_5, 12_) = 5.033, *p* = 0.0102; *Per2*: F (_5, 12_) = 3.132, *p* = 0.0488; *Per3*: F (_5, 12_) = 3.355, *p* = 0.0398; *Rorβ*: F (_5, 12_) = 5.352, *p* = 0.0081; *Rev-erbα*: F (_5, 12_) = 38.67, *p* = 0.0001). Cosine analysis showed that all nine clock gene expression patterns showed significant circadian rhythms (Table 2).

**Figure 1:**
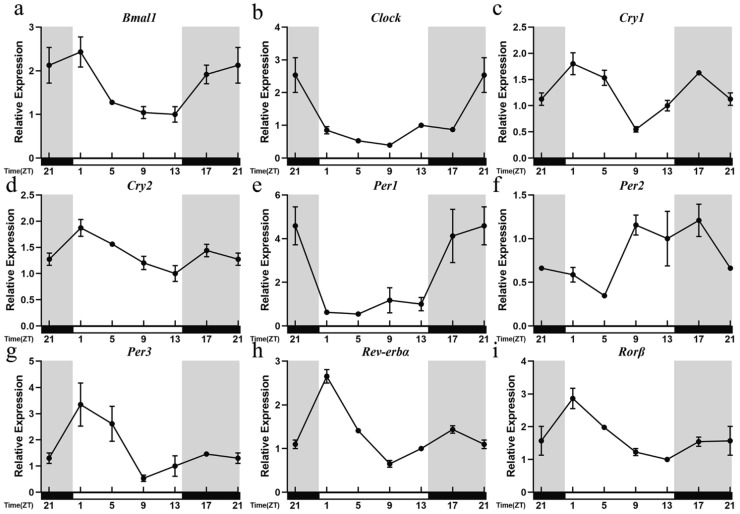
Temporal patterns of the mRNA levels of nine clock genes in the hypothalamus of mice in CON group. (**a**) *Bmal1*, (**b**) *Clock*, (**c**) *Cry1*, (**d**) *Cry2*, (**e**) *Per1*, (**f**) *Per2*, (**g**) *Per3*, (**h**) *Rev-erbα*, (**i**) *Rorβ*. The horizontal white bar on each figure represents the subjective daytime, and the black bar represents the subjective night. The relative mRNA levels were normalized to *Gapdh* and presented as the fold of ZT13; n = 3 mice per time point. Quantitative analysis of the PCR data is shown as the mean ± SEM. One-way ANOVA was used to evaluate the significance of differences among the six time points of the daily profile.

The peaks of positive clock genes *Bmal1* and *Clock* both appeared in the subjective night, while troughs all appeared in the subjective daytime (*Bmal1*: R^2^ = 0.90, *p* = 0.00; *Clock*: R^2^ = 0.58, *p* = 0.00). The acrophase of *Bmal1* appeared at the end of the subjective night and showed a downward trend in the subjective daytime (Figure 1a). The acrophase of *Clock* appeared at the beginning of the subjective night and showed an upward trend in the subjective daytime (Figure 1b).

Negative clock genes *Cry1* and *Cry2* showed a rhythm pattern that gradually decreased during the subjective daytime (*Cry1*: R^2^ = 0.43, *p* = 0.04; *Cry2*: R^2^ = 0.67, *p* = 0.00). Although the acrophase of *Cry1* appeared in the subjective night (ZT22.89), the acrophase of *Cry2* appeared in the subjective daytime (ZT1.32). They both reached the highest value at the beginning of the subjective daytime and were at a low level at the end (Figure 1c,d).

*Per1* and *Per2*, both negative clock genes, showed a rhythm pattern that gradually increased during the subjective daytime (*Per1*: R^2^ = 0.73, *p* = 0.00; *Per2*: R^2^ = 0.73, *p* = 0.00). Their acrophases all appeared in the subjective night (Figure 1e,f). However, *Per3* had a different time mode (*Per3*: R^2^ = 0.66, *p* = 0.00). It showed a tendency to gradually decrease during the subjective daytime, and its acrophase appeared at the beginning of the light phase (Figure 1g).

Branch feedback clock genes *Rorβ* and *Rev-erbα* both showed a tendency to gradually decrease during the subjective daytime (*Rorβ*: R^2^ = 0.77, *p* = 0.00; *Rev-erbα*: R^2^ = 0.51, *p* = 0.01). Their acrophases appeared at the beginning of the subjective daytime and were minimized at the beginning of the subjective night (Figure 1h,i). The acrophases of nine clock genes were all in the subjective night and at the end of the subjective night.

### 3.2. Circadian Rhythm Changes of Hypothalamic Positive Clock Genes after Sleep Restriction

**Figure 2:**
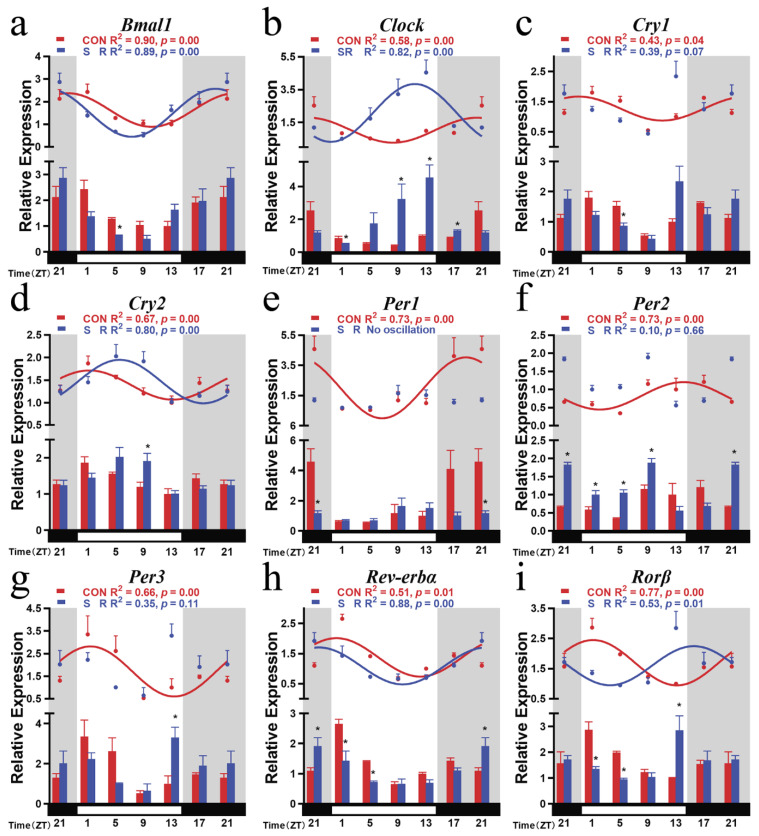
Temporal changes in the mRNA levels of nine clock genes in the hypothalamus of mice in SR group. (**a**) *Bmal1*, (**b**) *Clock*, (**c**) *Cry1*, (**d**) *Cry2*, (**e**) *Per1*, (**f**) *Per2*, (**g**) *Per3*, (**h**) *Rev-erbα*, (**i**) *Rorβ*. The horizontal white bar on each figure represents the subjective daytime, and the black bar represents the subjective night. The relative mRNA levels were normalized to *Gapdh* and presented as the fold of ZT13; n = 3 mice per time point. Quantitative analysis of the PCR data is shown as the mean ± SEM. The curve indicates the best fit to the points by cosinor analysis. R^2^ values represent the degree of fitting. *: *p*-values indicate the significance of regression analysis, with significance defined as *p* < 0.05. The comparison at each time point uses *t*-tests.

After sleep restriction, positive clock genes *Bmal1* and *Clock* both had obvious circadian rhythm oscillations (*Bmal1*: F (_5, 12_) = 6.609, *p* = 0.0036; *Clock*: F (_5, 12_) = 4.860, *p* = 0.0116). Cosine analysis showed that they also had a circadian rhythm (*Bmal1*: R^2^ = 0.89, *p* = 0.00; *Clock*: R^2^ = 0.82, *p* = 0.00). Compared with the CON group, the trend of *Bmal1* overtime did not change, and its mesor and amplitude did not change, but its acrophase was advanced by 2.94 h after sleep restriction (Table 2; Figure 2a). For a single time point comparison, the *Bmal1* expression level of the sleep restriction group was significantly reduced at ZT5 in the subjective daytime (Figure 2a).

The amplitude of *Clock* increased significantly after sleep restriction treatment. The amplitude of the SR group was twice the amplitude of the CON group. The sleep restriction treatment also advanced the acrophase of *Clock* by 8.22 h, appearing in ZT11.80 in the subjective daytime. In comparing a single time point, the expression levels of ZT9, ZT13, and ZT17 all increased. The expression levels of ZT1 decreased (Table 2; Figure 2b).

### 3.3. Circadian Rhythm Changes of Hypothalamic Negative Clock Genes after Sleep Restriction

Compared with other clock genes, negative clock genes showed more obvious changes after sleep restriction. Among them, *Per1* lost its oscillation overtime after sleep restriction (*Per1*: F (_5, 12_) = 1.325, *p* = 0.3179; Figure 2e). Although *Cry1* and *Per2/3* still had time-varying oscillations (*Cry1*: F (_5, 12_) = 4.263, *p* = 0.0184; *Per2*: F (_5, 12_) = 23.66, *p* = 0.0001; *Per3*: F (_5, 12_) = 3.214, *p* = 0.0453), cosine analysis showed that they no longer had a 24 h circadian rhythm (*Cry1*: R^2^ = 0.39, *p* = 0.07; *Per2*: R^2^ = 0.10, *p* = 0.66; *Per3*: R^2^ = 0.35, *p* = 0.11). From the comparison at a single time point, *Cry1* was significantly lower than the CON group at ZT5; *Per1* was significantly lower at ZT21 than the CON group; *Per2* was significantly higher than the CON group at ZT21–ZT9 at four time points; *Per3* was significantly higher at ZT13 than the CON group (Figure 2c,e–g).

*Cry2* was the only negative clock gene present after sleep restriction (*Cry2*: R^2^ = 0.80, *p* = 0.00). It was still manifest in the temporal trend of declining in subjective daytime and increasing in subjective night (Figure 2d). However, after the sleep restriction treatment, its acrophase changed from ZT1.32 to ZT5.62 and was delayed. Its mesor and amplitude did not change (Table 2). From the change at a single time point, *Cry2* was significantly higher at ZT9 than the CON group.

Negative regulator clock genes showed a more sensitive response to reduced sleep time. After being treated with sleep restriction, their circadian rhythm changed more drastically.

### 3.4. Circadian Rhythm Changes of Hypothalamic Branch Feedback Clock Genes after Sleep Restriction

After sleep restriction treatment, *Rorβ* and *Rev-erbα* still showed significant time-varying oscillations (*Rorβ*: F (_5, 12_) = 3.759, *p* = 0.0280; *Rev-erb*α: F (_5, 12_) = 4.527, *p* = 0.0150). Cosine analysis showed both circadian rhythms with a 24 h cycle (*Rorβ*: R^2^ = 0.53, *p* = 0.01; *Rev-erbα*: R^2^ = 0.88, *p* = 0.00). However, their acrophases were all advanced (Figure 2h,i). *Rev-erbα* maintained the same time trend as that of the CON group. Its mesor changed to 1.0924, the acrophase changed to ZT21.49, and its amplitude did not change (Table 2; Figure 2h). At the same time, sleep restriction increased the expression of *Rev-erbα* in the subjective night of ZT21 and decreased the expression at ZT1 and ZT5 in the subjective daytime.

The acrophase of *Rorβ* was advanced by 9.78 h, and the trend over time also changed (Figure 2i). The SR group showed a gradual increase in the subjective daytime, with a peak at ZT15.71; then, it gradually decreased in the subjective night. Sleep restriction also reduced the expression at ZT1 and ZT5 of *Rorβ* during the subjective night and, at the same time, increased the expression at ZT13 at the end of the subjective daytime (Table 2; Figure 2i). *Rev-erbα* and *Rorβ* showed similar trends in the SR group.

### 3.5. Circadian Rhythm Changes of Hypothalamic Clock Protein PER2 and Upstream Signals after Sleep Restriction

**Figure 3:**
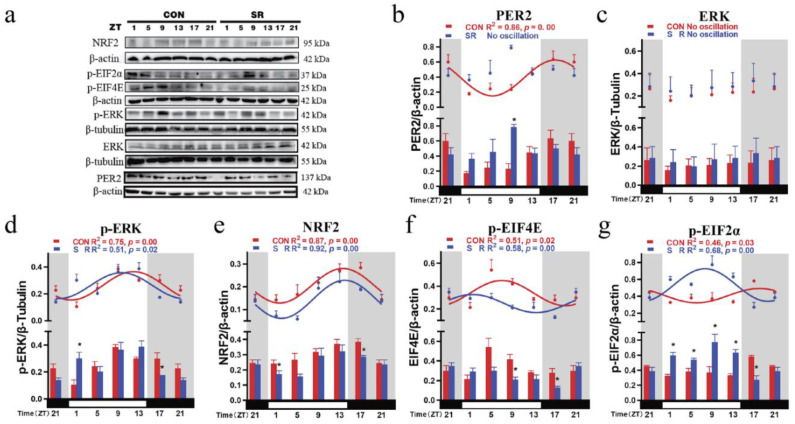
Temporal changes in the levels of proteins in the hypothalamus of mice. (**a**) PER2, ERK, p-ERK, NRF2, p-EIF4E, p-EIF2α, β-actin, and β-tubulin protein production in the CON and SR groups was examined by Western blotting, and relative protein levels of NRF2, p-EIF4E, and p-EIF2α were normalized to β-actin, while relative protein levels of ERK and p-ERK were normalized to β-tubulin. (**b**–**f**) The trend of PER2, ERK, p-ERK, NRF2, p-EIF4E, and p-EIF2α over time and the comparison at a specific point in time. The horizontal white bar on each figure represents the subjective daytime, and the black bar represents the subjective night; n = 3 mice per time point. Quantitative analysis of the data is shown as the mean ± SEM. The curve indicates the best fit to the points by cosinor analysis. R^2^ values represent the degree of fitting. *: *p*-values indicate the significance of regression analysis, with significance defined as *p* < 0.05. The comparison at each time point uses *t*-tests.

Clock protein PER2 had a 24 h circadian rhythm in the CON group (PER2: F (_5, 12_) = 4.058, *p* = 0.0218, R^2^ = 0.86, *p* = 0.00; Table 1; Table 2). Similar to the mRNA results, the acrophase of clock proteins was also at the subjective night and the beginning of subjective day (Figure 3b). Compared with the acrophase of mRNA, the acrophase of PER2 was 4.26 h away from the acrophase of mRNA (*p* = 0.0453). In the SR group, the negative regulatory clock gene PER2 disappeared after sleep restriction (PER2: F (_5, 12_) = 2.313, *p* = 0.1086; Table 1 and Table 2).

PER2 reached a valley in the early subjective daytime in the CON group; then, it gradually increased and reached a peak in the subjective early night. After sleep restriction, the oscillation of PER2 over time disappeared, and the circadian rhythm was lost. At the same time, after sleep restriction, PER2 increased significantly during the subjective daytime ZT9 (Figure 3b).

ERK did not show oscillations over time in the hypothalamus (CON: F (_5, 12_) = 0.098, *p* = 0.9906; SR: F (_5, 12_) = 0.078, *p* = 0.9945; Table 1). However, p-ERK had a circadian rhythm with a 24 h cycle (CON: F (_5, 12_) = 5.960, *p* = 0.0054, R^2^ = 0.75, *p* = 0.00; SR: F (_5, 12_) = 4.978, *p* = 0.0107, R^2^ = 0.51, *p* = 0.02). It was at a valley at the beginning of the subjective daytime then gradually rose, reaching a peak at the end of the subjective daytime. Sleep restriction advanced the phase of the p-ERK peak, which appeared in the middle of the subjective daytime (Figure 3d). At the same time, changes were the most obvious at the moment of light switching, such as ZT1 and ZT17 (Figure 3d).

NRF2 in the hypothalamus had a 24 h circadian rhythm in the CON and SR groups (CON: F (_5, 12_) = 3.352, *p* = 0.0399, R^2^ = 0.87, *p* = 0.00; SR: F (_5, 12_) = 4.207, *p* = 0.0193, R^2^ = 0.92, *p* = 0.00). It reached a valley at the beginning of the subjective daytime then gradually rose, reaching a peak at the beginning of the subjective night. Sleep restriction did not change its trend over time (Figure 3e). However, sleep restriction reduced the mesor value of NRF2 (Table 2). Moreover, the decrease was most obvious at the moment of light switching (Figure 3e). The mesor value of NRF2 was positively correlated with the mesor values of *Rev-erbα* (r^2^ = 0.84, *p* = 0.01).

p-EIF4E had a 24 h circadian rhythm in both the CON group and the SR group (CON: F (_5, 12_) = 3.378, *p* = 0.0390, R^2^ = 0.51, *p* = 0.02; SR: F (_5, 12_) = 3.850, *p* = 0.0259, R^2^ = 0.58, *p* = 0.00). In the CON group, p-EIF4E peaked in the middle of the subjective daytime and remained low during the subjective night. The sleep restriction caused its acrophase to move to the beginning of the subjective daytime and reach the valley at the end of the subjective daytime. The sleep restriction decreased the expression at ZT9 and ZT17 (Figure 3f). p-EIF4E was positively correlated with the acrophases of clock genes *Bmal1* (r^2^ = 0.99, *p* = 0.00), *Clock* (r^2^ = 0.93, *p* = 0.00), *Rev-erbα* (r^2^ = 0.67, *p* = 0.04), and *Rorβ* (r^2^ = 0.98, *p* = 0.00).

p-EIF2α also had a 24 h circadian rhythm in the CON and SR groups (CON: F (_5, 12_) = 4.092, *p* = 0.0212, R^2^ = 0.46, *p* = 0.03; SR: F (_5, 12_) = 6.509, *p* = 0.0038, R^2^ = 0.68, *p* = 0.00). In the CON group, p-EIF2α peaked in the subjective early night, while the valley appeared in the middle of the subjective daytime. After sleep restriction, its peak appeared in the middle of the subjective daytime. The sleep restriction increased the expression at ZT1–ZT13 during the subjective daytime and decreased the expression at ZT17 during the subjective night (Figure 3g). Its mesor and amplitude also increased significantly after sleep restriction (Table 2).

p-EIF2α was positively correlated with the acrophase of the *Bmal1* (r^2^ = 0.88, *p* = 0.00), *Clock* (r^2^ = 0.79, *p* = 0.02), *Rev-erbα* (r^2^ = 0.76, *p* = 0.02), and *Rorβ* (r^2^ = 0.99, *p* = 0.00) and negatively correlated with clock gene *Cry2* (r^2^ = 0.76, *p* = 0.03). p-EIF2α showed a negatively correlated mesor change with *Rev-erbα* (r^2^ = 0.89, *p* = 0.00). p-EIF2α showed an amplitude change positively correlated with *Clock* (r^2^ = 0.81, *p* = 0.01).

### 3.6. Circadian Rhythm Changes of Plasma Melatonin, Corticosterone, IL-6, and TNF-α after Sleep Restriction

**Figure 4:**
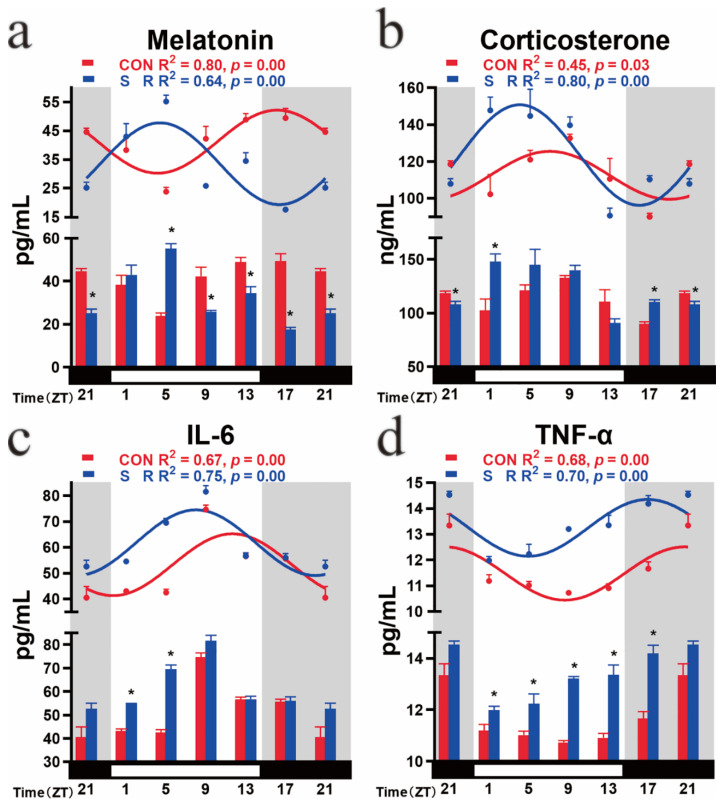
Temporal changes in the plasma melatonin, corticosterone, IL-6, and TNF-α concentrations of mice in the different groups; (**a**) melatonin, (**b**) corticosterone, (**c**) IL-6, (**d**) TNF-α. The horizontal white bar on each figure represents the subjective day, and the black bar represents the subjective night; n = 3 mice per time point. The data are shown as the mean ± SEM. The curve indicates the best fit to the points by cosinor analysis. R^2^ values represent the degree of fitting. *: *p*-values indicate the significance of regression analysis, with significance defined as *p* < 0.05. The comparison at each time point uses *t*-tests.

In the CON group, plasma melatonin, corticosterone, IL-6, and TNF-α all exhibited oscillations over time (melatonin: F (_5, 12_) = 6.303, *p* = 0.0043; corticosterone: F (_5, 12_) = 3.286, *p* = 0.0424; IL-6: F (_5, 12_) = 25.78, *p* = 0.0000; TNF-α: F (_5, 12_) = 10.03, *p* = 0.0006). The cosine analysis showed that all four had a 24 h cycle of circadian rhythm changes (melatonin: R^2^ = 0.80, *p* = 0.00; corticosterone: R^2^ = 0.45, *p* = 0.03; IL-6: R^2^ = 0.67, *p* = 0.00; TNF-α: R^2^ = 0.68, *p* = 0.00). Plasma melatonin gradually decreased with light and reached a trough at ZT4.06; then, it increased, reached a peak at ZT16.06, and maintained high secretion in the dark (Figure 4a). Plasma corticosterone increased during the light phase, peaked at ZT6.86, and remained low during the subjective night (Figure 4b). IL-6 remained low in the early stage of light and then gradually increased, reaching a peak at ZT11.29 and maintaining a low level at the subjective night (Figure 4c). TNF-α remained low during the subjective daytime, reached a trough at ZT8.61, and then remained high during the subjective night (Figure 4d).

After the sleep restriction treatment, the changing trend of the plasma melatonin of mice over time showed an opposite trend to that of the CON group. It reached a peak under light conditions at ZT4.48 then gradually decreased and reached a trough under dark conditions. The acrophase time of the SR group was advanced by 11.58 h. In addition, the mesor of the SR group was significantly lower than that of the CON group by 18.67%; there was no change in the amplitude. From the comparison at a single time point, sleep restriction caused melatonin to increase early in the subjective daytime and then decrease compared to the CON group (Figure 4a).

The mesor plasma corticosterone increased after sleep restriction. Comparing a single time point, the secretion of ZT1 and ZT17 increased significantly after sleep restriction, and ZT21 decreased significantly after sleep restriction (Figure 4b).

Plasma proinflammatory factors IL-6 and TNF-α showed the same change trend after sleep restriction. Their acrophases were all advanced (Figure 4c,d). At the same time, their mesor had also risen significantly. Considering the change at a single time point, sleep restriction mainly caused the increase of both in the subjective daytime; the increase of IL-6 was mainly in ZT1 and ZT5, and the increase of TNF-α was in ZT1–ZT17 (Figure 4c,d). Sleep restriction increases the secretion of inflammatory factors.

The three rhythm output signals (Mel, IL6, and TNFα) whose acrophases changed all showed phase changes that were positively correlated with positive clock genes (IL6: *Bmal1*, R^2^ = 0.90, *p* = 0.00; *Clock*, R^2^ = 0.96, *p* = 0.00; Mel: *Bmal1*, R^2^ = 0.94, *p* = 0.00; *Clock*, R^2^ = 0.96, *p* = 0.00; TNFα: *Bmal1*, R^2^ = 0.88, *p* = 0.01; *Clock*, R^2^ = 0.96, *p* = 0.00) and branch feedback clock genes (IL6: *Rev-erbα*, R^2^ = 0.68, *p* = 0.04; *Rorβ*, R^2^ = 0.96, *p* = 0.00; Mel: *Rev-erbα*, R^2^ = 0.70, *p* = 0.04; *Rorβ*, R^2^ = 0.96, *p* = 0.00; TNFα: *Rev-erbα*, R^2^ = 0.68, *p* = 0.04; *Rorβ*, R^2^ = 0.96, *p* = 0.00) and negatively correlated with negative clock genes (IL6: *Cry2*, R^2^ = 0.85, *p* = 0.01; Mel: *Cry2*, R^2^ = 0.76, *p* = 0.03; TNFα: *Cry2*, R^2^ = 0.86, *p* = 0.01). In the output signals whose mesor value changed, TNFα showed a negatively correlated mesor value change with *Rev-erbα* (R^2^ = 0.92, *p* = 0.00). Melatonin showed a positive correlation with *Rev-erbα* in the mesor change (R^2^ = 0.82, *p* = 0.01).

## 4. Discussion

In this study, we confirmed the existence of circadian rhythms in nine clock genes (*Bmal1*, *Clock*, *Cry1*, *Cry2*, *Per1*, *Per2*, *Per3*, *Rev-erbα*, and *Rorβ*) in the mouse hypothalamus. *Bmal1*, *Clock*, *Per2*, *Per3*, and *Rev-erbα* were consistent with previously reported rhythm patterns in mouse SCN [38,39,40,41]. *Per1*, *Cry1*, and *Cry2* are different from previous reports. Our results showed that *Per1* is low in the subjective daytime, while previous studies showed that *Per1* is low in the subjective night [11] and that *Cry1* tends to increase during the subjective daytime; however, our results show a downward trend during the subjective daytime [42]. *Cry2* used to have no apparent circadian rhythm, but our results detected a 24 h cycle of the circadian rhythm [42]. However, compared to other clock genes, *Cry2* has the smallest amplitude. These differences may be due to different factors, such as animal strains, technical methods, and seasons. *Rorβ* is hardly reported in the detection of circadian rhythms in mouse SCN. However, our results are consistent with the expression of RORβ in rat SCN [43]. This difference reflects the tendency of the circadian clock system to show diversity in different species and strains with the time change.

Our results showed that sleep restriction had a disruptive effect on the circadian rhythm of the clock gene expression in the hypothalamus, which was particularly evident in the negative clock genes. The circadian rhythms of negative clock genes *Cry1* and *Per1/2/3* disappeared due to sleep restriction treatment. According to our results, the circadian rhythm of the *Per* gene was also sensitive to sleep reduction. *Per2* and *Per3* are related to and regulate sleep [44,45]. Previous studies at a single time point found that 6 h of sleep deprivation can increase the levels of *Per1* and *Per2* mRNA in the cerebral cortex of mice [18]. Our results indicate that changes in sleep can also reversely regulate the circadian rhythm of the *Per* gene. Abnormality of the *Per1* gene can lead to changes in the balance of apoptosis and proliferation and has the risk of causing cancer [46,47]. Since *Per1/2/3* genes play a considerable role in maintaining rhythm, the disappearance of their circadian rhythm may cause potential negative effects on the body.

Clock genes that still maintained the circadian rhythm after sleep restriction treatment include positive clock gene *Bmal1/Clock*, branch-regulating gene *Rorβ/Rev-erbα*, and negative clock gene *Cry2*. Although other clock genes still had circadian rhythms, their mesor, amplitudes, and acrophases also changed more or less, due to sleep restriction processing. Sleep restriction had a universal effect, at least on the circadian rhythm of clock gene expression. The disappearance of the rhythm of the *Bmal1* gene disrupts an animal’s rhythmic behavior and leads to an increase in sleep and the ability to recover from lack of sleep [48,49]. At the same time, an interesting study showed that restoring the *Bmal1* rhythm in the muscles but not in the brain can restore sleep in mice [50]. Previous studies also found that mutations in the *Clock* gene reduce rest time and cause changes in response to sleep deprivation [51,52]. These phenomena all indicate the close relationship between the positive clock gene and sleep. The acrophase of the positive regulation element after sleep restriction may be related to the early end of sleep time. An exception to the negative clock gene was *Cry2*, which maintained the circadian rhythm after sleep restriction. The *Cry* gene was confirmed to be connected with sleep [18]. Mutations in the human *Cry* gene can cause sleep disorders characterized by late sleep [53]. However, there were also differences between CRY1 and CRY2. The critical difference between them was the basis of their differential strength as transcription repressors. Both proteins bind to BMAL1 and inhibit CLOCK:BMAL1 activity. However, CRY1 has a higher affinity with CLOCK:BMAL1, so that it can act as a more potent repressor and extend the day–night cycle [54]. The different circadian cycles of *Cry1*-deficient and *Cry2*-deficient mice also reflect their different roles in the biological clock [55]. At the same time, CRY is also considered a blue light-sensitive photopigment in mice [56]. Previous research in our laboratory also found that *Cry2* is highly sensitive to red light in chickens [57]. Sleep restriction is non-light treatment. The rhythm that *Cry2* still exhibits after treatment may indicate that it is more sensitive to changes in light than the reduction in sleep time. *Rev-erbα* knockout causes mice to more slowly increase their sleep requirements [58]. Furthermore, sleep deprivation can lead to a decrease in *Rev-erbα* expression [59]. Similar to previous studies, our results also showed that the mesor value of *Rev-erbα* reduction after sleep time was reduced. RORβ is mainly expressed in the central nervous system, especially in the SCN, which participates in the circadian rhythm [60,61]. The rhythmic performance of *Rorβ* after sleep restriction has not been reported. However, a large-scale population survey showed that the interaction between RORA and RORB affects the duration of sleep [62]. Our results indicate that *Rorβ* is regulated by sleep duration and may play a unique role in sleep. Since the clock genes mentioned above have other functions besides participating in the circadian rhythm, the loss and change of their circadian rhythm may affect many aspects of the body and cause adverse effects.

There are many kinds of cells with self-pacing ability in the hypothalamus. Vasopressin (AVP) and vasoactive intestinal peptide (VIP) neurons in SCN have different network synchrony and stability [63]. SCN astrocytes can also drive the circadian rhythm of mammals [64]. The sensitivity of different types of cells to sleep restriction is likely different. Therefore, the classification of different cells is greatly valuable.

The MAPK pathway is upstream of a biological clock that can transmit light signals [65,66]. Similar to previous findings, under normal circumstances, the activity of ERK in the mouse hypothalamus was high in the subjective daytime and low in the subjective night [67,68,69]. When Syrian hamsters suffer from short-term sleep deprivation, ERK activity in SCN is inhibited [23]. Our results indicate that long-term sleep restriction can cause p-ERK phase shifts in the mouse hypothalamus. This result proves that long-term sleep restriction can also affect the normal rhythmic activation of ERK and indicates that the length of sleep deprivation affects the difference in ERK activity.

ERK is involved in the activation of NRF2 and the signal transduction mediated by NRF2 [70,71]. ERK inhibition reduces the protein stability of NRF2, promotes its degradation, and reduces its nuclear accumulation [72]. In our results, the advance of the acrophase of phosphorylated ERK led to the reduction of NRF2. Moreover, NRF2 is a transcription factor that modulates endogenous antioxidants and antioxidant enzymes [73]. Therefore, SR could lead to a decrease in the antioxidant capacity of the hypothalamus. Recent studies also showed that NRF2 participates in multiple links in the circadian rhythm and regulates the expression of *Per3* and *Rev-erbα*. Our results also show that the mesor changes of NRF2 and *Rev-erbα* show a positive correlation trend. Together, NRF2 is a critical node linking metabolism to the clock and a conduit for the response to changes in intracellular redox status [25]. The above data and results also imply that the destruction of the central biological clock by SR may be achieved through oxidative stress.

The ERK pathway also mediates the rhythmic regulation of light signals. The ERK pathway leads to EIF4E phosphorylation through the MAPK-interacting serine/threonine-protein kinases (MNKs) in the photorecipient SCN cells and facilitates light-induced PER protein synthesis. Circadian clock-regulated EIF4E phosphorylation promotes basal PER protein synthesis [28]. We obtained p-EIF4E circadian rhythm trends similar to those of a previous study [28]. The decrease in the level of p-EIF4E is also consistent with the reduction in the level of *Per1*.

The level of phosphorylated EIF2α determines the cell’s response to stress and participates in endoplasmic reticulum stress [74,75]. EIF2α is involved in the regulation of the biological clock [29]. The phosphorylation of EIF2α activates the mRNA translation of the transcriptional regulator of transcription factor 4 (*Atf4*) to regulate the biological clock and promote *Per2* transcription [76]. The phosphorylation of EIF2α occurs in the integrated stress response (ISR), which suggests that the disorder of EIF2α may be the basis of neurological diseases with circadian rhythm dysfunction [75]. We obtained p-EIF2α circadian rhythm trends similar to the previous study [29]. In the long-term lack of sleep, we also found the enhancement of EIF2α phosphorylation and the increase of *Per2* mRNA and protein levels. The profound changes in its amplitude and acrophase may also cause *Per2′*s loss of circadian rhythm. The altered PER2 further affects the positive clock gene. The above results found that, although sleep restriction is a non-light treatment, it also changes the animal’s perception of light. Changes in EIF2α also suggest that SR can cause endoplasmic reticulum stress in the central circadian clock.

Sleep restriction leads to disturbance of the circadian rhythm of the central biological clock in the hypothalamus, which affects the rhythm of its output signal. Since pineal melatonin is synthesized and released into the bloodstream under the control of the endogenous clock in the SCN [77], the disrupted clock gene circadian rhythm makes a significant change in the circadian rhythm of melatonin. This is similar to delayed dim-light melatonin onset caused in sleep disorders [78,79]. The circadian rhythm of corticosterone was mainly reflected in the increase in the mesor value. This suggests that the body’s stress state caused by sleep restriction treatment may be the result of the SCN circadian clock rhythm disorder. This phenomenon helps explain obesity and diabetes caused by abnormal rhythms such as shifts [80].

As in previous reports, both IL-6 and TNF-α showed high levels after reduced sleep time [35,81]. Their acrophases were all advanced, which was consistent with the trend of the acrophase shift of melatonin. At the same time, their mesor showed the opposite trend to that of melatonin. Previous studies showed that melatonin can inhibit the synthesis of IL-6 and TNF-α [82,83]. An elevated mesor indicates the occurrence of inflammation in the body. The advanced acrophase indicates a disorder of the normal rhythm of the immune system. This may be one of the reasons for the body’s immune function damage and disease caused by lack of sleep.

## 5. Conclusions

Long-term lack of sleep can cause rhythm disorder of the upstream regulatory factors of the biological clock, further cause disturbance of the circadian rhythm of the hypothalamic core pacemaker, and damage the antioxidant function of the hypothalamus. It also changes the circadian pattern of output signals melatonin and corticosterone regulated by the hypothalamus, which promotes abnormality in—and organization of systemic damage to—immune function. The results of this study also provide new ideas and directions for improving body damage caused by long-term lack of sleep.

## Figures and Tables

**Table 1:** Primers used for real-time PCR analysis and expected product length.

Gene	Accession No.	Primer Sequence (5′ to 3′)		Length (bp)
*Bmal1*	NM_001374642.1	F:CAGAGCCGGAGCAGGAAAAATAGGT	R:CAGGGGGAGGCGTACTTGTGATGT	128
*Clock*	XM_011249402.2	F: ATGGTGTTTACCGTAAGCTGTAG	R: CTCGCGTTACCAGGAAGCAT	197
*Cry1*	NM_007771.3	F: CACTGGTTCCGAAAGGGACTC	R: CTGAAGCAAAAATCGCCACCT	153
*Cry2*	NM_009963.4	F: CACTGGTTCCGCAAAGGACTA	R: CCACGGGTCGAGGATGTAGA	102
*Per1*	NM_001159367.2	F: CGGATTGTCTATATTTCGGAGCA	R: TGGGCAGTCGAGATGGTGTA	142
*Per2*	NM_011066.3	F: GAAAGCTGTCACCACCATAGAA	R: AACTCGCACTTCCTTTTCAGG	186
*Per3*	NM_001289878.1	F: TCAAGACGTGAGGGCGTTCTA	R: CATTCATACTGCGAGGCTCTTT	90
*Rorβ*	NM_001289921.1	F: GCAGCATTAGCAATGGCCTC	R: GACGGCTGACCGGAATCTATG	121
*Reverbα*	NM_145434.4	F: TACATTGGCTCTAGTGGCTCC	R: CAGTAGGTGATGGTGGGAAGTA	127
*Gapdh*	NM_001289726.1	F: CCGAGAATGGGAAGCTTGTC	R: TTCTCGTGGTTCACACCCATC	232

F = forward primer; R = reverse primer.

**Table 2:** Rhythm parameters (mean ± SEM) of genes, proteins, and plasma rhythm output signal in mice under control group and sleep restriction group (14 L:10 D), as determined by cosinor analysis.

	Mesor		Amplitude		Acrophase (ZT)	
	SR	CON	SR	CON	SR	CON
*Bmal1*	1.51 ± 0.23	1.63 ± 0.21	1.07 ± 0.20	0.75 ± 0.14	19.47 ± 0.23 *	22.28 ± 0.44
*Clock*	2.08 ± 0.40	1.03 ± 0.13	1.85 ± 0.34 *	0.77 ± 0.16	11.80 ± 0.82 *	20.02 ± 0.41
*Cry1*	-	1.27 ± 0.11	-	0.41 ± 0.041	-	22.89 ± 0.58
*Cry2*	1.47 ± 0.10	1.391 ± 0.04	0.49 ± 0.11	0.33 ± 0.05	5.62 ± 0.36 *	1.32 ± 0.39
*Per1*	-	2.01 ± 0.48	-	2.06 ± 0.41	-	19.05 ± 0.55
*Per2*	-	0.83 ± 0.08	-	0.40 ± 0.09	-	14.09 ± 0.75
*Per3*	-	1.71 ± 0.36	-	1.14 ± 0.31	-	1.38 ± 0.59
*Rorβ*	1.60 ±0.03	1.70 ± 0.15	0.72 ± 0.06	0.79 ± 0.13	15.71 ± 1.00 *	1.49 ± 0.73
*Reverbα*	1.09 ± 0.06 *	1.38 ± 0.01	0.66 ± 0.06	0.64 ± 0.11	21.49 ± 0.78 *	0.13 ± 0.21
BMAL1	0.56 ± 0.06	0.58 ± 0.07	0.24 ± 0.01 *	0.27 ± 0.00	8.15 ± 0.42 *	20.31 ± 0.62
CLOCK	0.22 ± 0.01 *	0.33 ± 0.04	0.09 ± 0.02	0.18 ± 0.02	3.44 ± 0.67 *	6.19 ± 0.32
PER2	-	0.39 ± 0.04	-	0.25 ± 0.07	-	18.35 ± 1.10
NRF2	0.24 ± 0.02 *	0.30 ± 0.01	0.10 ± 0.01	0.09 ± 0.01	13.98 ± 0.68	14.15 ± 1.19
p-ERK	0.26 ± 0.03	0.26 ± 0.03	0.10 ± 0.02	0.11 ± 0.01	9.81 ± 0.70 *	11.92 ± 0.19
p-EIF4E	0.25 ± 0.03	0.34 ± 0.05	0.08 ± 0.00 *	0.12 ± 0.01	1.52 ± 0.49 *	7.12 ± 0.72
p-EIF2α	0.53 ± 0.04 *	0.41 ± 0.02	0.20 ± 0.02 *	0.10 ± 0.02	7.54 ± 0.70 *	14.71 ± 0.78
Melatonin	33.55 ± 1.23 *	41.25 ± 0.85	14.42 ± 1.35	12.61 ± 0.17	4.48 ± 0.34 *	16.06 ± 1.14
Corticosterone	123.58 ± 1.86 *	112.56 ± 2.00	28.11 ± 3.50	16.49 ± 4.56	3.94 ± 0.58	6.86 ± 1.26
IL-6	61.85 ± 1.56 *	52.19 ± 1.61	12.72 ± 0.22	13.78 ± 1.09	7.99 ± 0.10 *	11.29 ± 0.11
TNF-α	13.25 ± 0.23 *	11.48 ± 0.17	1.10 ± 0.05	1.03 ± 0.10	16.96 ± 0.16 *	20.61 ± 0.03

*: Significant differences between different groups (*p* < 0.05).

## Data Availability

The data presented in this study are available on request from the corresponding author.
